# Predicting acute uncomplicated urinary tract infection in women: a systematic review of the diagnostic accuracy of symptoms and signs

**DOI:** 10.1186/1471-2296-11-78

**Published:** 2010-10-24

**Authors:** Leonie GM Giesen, Gráinne Cousins, Borislav D Dimitrov, Floris A van de Laar, Tom Fahey

**Affiliations:** 1 HRB Centre for Primary Care Research, Department of General Practice, Royal College of Surgeons in Ireland, Beaux Lane House, Lower Mercer Street, Dublin 2, Ireland; 2 Department of Primary and Community Care, Radboud University Nijmegen Medical Centre, 6500 HB, Nijmegen, The Netherlands

## Abstract

**Background:**

Acute urinary tract infections (UTI) are one of the most common bacterial infections among women presenting to primary care. However, there is a lack of consensus regarding the optimal reference standard threshold for diagnosing UTI. The objective of this systematic review is to determine the diagnostic accuracy of symptoms and signs in women presenting with suspected UTI, across three different reference standards (10^2 ^or 10^3 ^or 10^5 ^CFU/ml). We also examine the diagnostic value of individual symptoms and signs combined with dipstick test results in terms of clinical decision making.

**Methods:**

Searches were performed through PubMed (1966 to April 2010), EMBASE (1973 to April 2010), Cochrane library (1973 to April 2010), Google scholar and reference checking.

Studies that assessed the diagnostic accuracy of symptoms and signs of an uncomplicated UTI using a urine culture from a clean-catch or catherised urine specimen as the reference standard, with a reference standard of at least ≥ 10^2 ^CFU/ml were included. Synthesised data from a high quality systematic review were used regarding dipstick results. Studies were combined using a bivariate random effects model.

**Results:**

Sixteen studies incorporating 3,711 patients are included. The weighted prior probability of UTI varies across diagnostic threshold, 65.1% at ≥ 10^2 ^CFU/ml; 55.4% at ≥ 10^3 ^CFU/ml and 44.8% at ≥ 10^2 ^CFU/ml ≥ 10^5 ^CFU/ml. Six symptoms are identified as useful diagnostic symptoms when a threshold of ≥ 10^2 ^CFU/ml is the reference standard. Presence of dysuria (+LR 1.30 95% CI 1.20-1.41), frequency (+LR 1.10 95% CI 1.04-1.16), hematuria (+LR 1.72 95%CI 1.30-2.27), nocturia (+LR 1.30 95% CI 1.08-1.56) and urgency (+LR 1.22 95% CI 1.11-1.34) all increase the probability of UTI. The presence of vaginal discharge (+LR 0.65 95% CI 0.51-0.83) decreases the probability of UTI. Presence of hematuria has the highest diagnostic utility, raising the post-test probability of UTI to 75.8% at ≥ 10^2 ^CFU/ml and 67.4% at ≥ 10^3 ^CFU/ml. Probability of UTI increases to 93.3% and 90.1% at ≥ 10^2 ^CFU/ml and ≥ 10^3 ^CFU/ml respectively when presence of hematuria is combined with a positive dipstick result for nitrites. Subgroup analysis shows improved diagnostic accuracy using lower reference standards ≥ 10^2 ^CFU/ml and ≥ 10^3 ^CFU/ml.

**Conclusions:**

Individual symptoms and signs have a modest ability to raise the pretest-risk of UTI. Diagnostic accuracy improves considerably when combined with dipstick tests particularly tests for nitrites.

## Background

Acute uncomplicated urinary tract infections (UTI) are one of the most common bacterial infections among women presenting to primary care, with an annual incidence of 7% for all ages of women peaking at 15-24 years and women older than 65 [1]. Approximately one third of all women have had at least one physician-diagnosed uncomplicated UTI by the age of 26 years [2].

The original reference standard for diagnosing UTI was the presence of significant bacteriuria, defined as the isolation of at least 10^5 ^colony-forming units (CFU) of a single uropathogen, in a clean catch or catherised urine specimen [3]. However, this cut-off limit has been debated in recent years resulting in the use of reduced diagnostic thresholds ranging from 10^2 ^[4-7] and 10^3 ^[8-11].

The pre-test probability of asymptomatic bacteriuria in women of reproductive age is approximately 5% [12,13]. However, the pre-test probability of an uncomplicated UTI is shown to increase from 5% to 50% among women presenting with at least one symptom of an uncomplicated UTI [14]. Symptoms of an uncomplicated UTI include dysuria (painful voiding), frequency (frequent voiding of urine), urgency (the urge to void immediately), and hematuria (presence of blood in urine). In contrast, patients presenting with vaginal discharge or irritation have a decreased risk of an uncomplicated UTI [14]. The presence or absence of symptoms function as useful diagnostic tests. Near patient testing in the form of urinary dipsticks are also commonly used in Primary Care to improve the precision of UTI diagnosis, providing immediate results which can be interpreted alongside patient symptoms.

Although empirical treatment of UTI is most cost-effective [15,16], prescribing without confirmation of diagnosis contributes to the growing problem of resistance against uropathogens in primary care [17].

A previous systematic review established the diagnostic accuracy of symptoms and signs for UTI [14], however, it remains unclear whether the diagnostic accuracy of symptoms and signs varies when alternative reference standards are applied. The aim of this systematic review is to determine the diagnostic accuracy of symptoms and signs of UTI in adult women across three different reference standards, 10^2^, 10^3 ^and 10^5 ^CFU/ml. In addition, we aim to determine the diagnostic accuracy of symptoms and signs combined with dipstick test results.

## Methods

The PRISMA guidelines for reporting on systematic reviews and meta-analysis were followed to conduct this review (Additional file 1).

### Search strategy

We performed a systematic search of three online databases, Pubmed (1966 to April 2010), Embase (1973 to April 2010) and the Cochrane Library (1973 to April 2010). A combination of MeSH terms and text words were used including: 'urinary tract infection/pyelonephritis/cystitis/urethritis', 'physical examination/medical history taking/professional competence', 'sensitivity and specificity', ' reproducibility of results/diagnostic tests, routine/decision support techniques/bayes theorem/predictive value of tests'. All combinations were restricted to 'women and female'. This search was supplemented by checking references of filtered papers and searching Google Scholar [18]. No restrictions were placed on language.

### Study selection

To be eligible for inclusion, the studies had to fulfil the following criteria:

1) Have a study population of adult symptomatic women with suspected uncomplicated UTI presenting to a primary care setting.

2) Use a cohort or cross-sectional study design. Case control studies were excluded.

3) Investigate the diagnostic accuracy of symptoms and signs of UTI using a urine culture from a clean-catch or catherised urine specimen as the reference test, with a diagnostic threshold of at least ≥ 10^2 ^CFU/ml.

4) Include sufficient data to allow for the calculation of sensitivity, specificity, negative and positive predictive values and the prevalence of uncomplicated UTI.

### Data extraction

The number of true positives, false positives, true negatives and false negatives for each sign and symptom were extracted from each of the studies and a 2 × 2 table was constructed. Discrepancies were resolved by discussion between the two reviewers (LG and GC). Authors were contacted to provide further information when there was insufficient detail in an article to construct a 2 × 2 table.

### Quality assessment

The methodological quality of the selected studies was evaluated independently by two reviewers (LG and GC) using the Quality Assessment of Diagnostic Accuracy Studies (QUADAS) tool, a validated tool for the quality assessment of diagnostic accuracy studies [19]. This tool was modified to ensure appropriateness to the present study and included twelve questions from the QUADAS tool with two additional questions extracted from a different review [20]. If no consensus was achieved, studies were evaluated by a third independent reviewer (TF).

### Data synthesis and analysis

#### Summary estimates across different reference standards

We used the bivariate random effects model to estimate summary estimates of sensitivity and specificity and their corresponding 95% confidence intervals. This approach was used as it preserves the two-dimensional nature of the original data and takes into account both study size and heterogeneity beyond chance between studies [21]. In addition, the bivariate model estimates and incorporates the negative correlation which may arise between the sensitivity and specificity of a given sign or symptom as a result of differences in reference standards used in different studies. These alternative thresholds are important when attempting to understand the diagnostic accuracy of symptoms and signs predicting uncomplicated UTI as studies have used different thresholds ranging from ≥ 10^2 ^CFU/ml, ≥ 10^3 ^CFU/ml and ≥ 10^5 ^CFU/ml. However, pooled estimates cannot be calculated using the bivariate model with less than 4 studies.

We plotted the individual and summary estimates of sensitivity and specificity for each symptom and sign at the different threshold levels in a receiver operating characteristic graph, plotting a symptom's sensitivity (true positive) on the y axis against 1-specificity (false negative) on the x axis. We also plotted the 95% confidence region and 95% prediction region around the pooled estimates to illustrate the precision with which the pooled values were estimated (confidence ellipse around the mean value) and to illustrate the amount of between study variation (prediction ellipse). We assessed heterogeneity visually using the summary ROC plots and statistically by using the variance of logit transformed sensitivity and specificity, with smaller values indicating less heterogeneity among studies.

#### Bayesian analysis and near patient testing (dipstick)

To examine the influence of threshold effects when considering alternative reference standards we conducted subgroup analysis across the three different thresholds: ≥ 10^2 ^CFU/ml, ≥ 10^3 ^CFU/ml and ≥ 10^5 ^CFU/ml. Using Bayes theorem the post-test odds of a UTI were estimated by multiplying the pretest odds by the likelihood ratio, where pre-test odds is calculated by dividing the pre-test probability by (1-pre-test probability) and the post-test probability equals post-test odds divided by (1 + post-test odds) [22]. Finally, the diagnostic accuracy of individual symptoms and signs combined with dipstick test results for nitrites, leucocyte-esterase and combined nitrites and leucocyte-esterase, was determined using data synthesised in a previous high quality systematic review regarding the diagnostic accuracy of dipstick urinalysis [23].

We used Stata version 10.1(StataCorp, College Station, Tx, USA), particularly the metandi commands, for all statistical analyses

## Results

### Search Strategy

**Figure 1:**
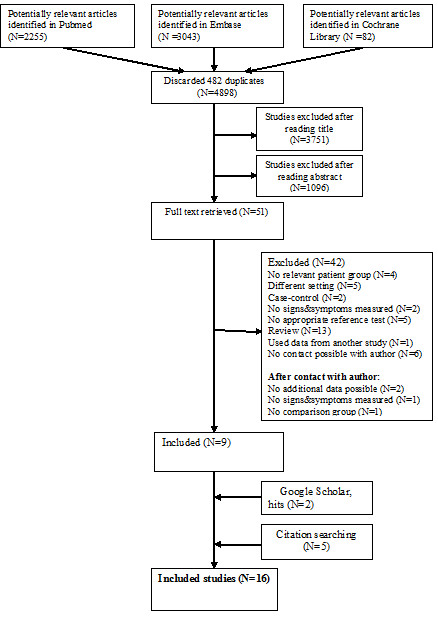
**Flow diagram of studies in the review**.

Two researchers (LG, GC) screened all potential articles and agreed that the full text of 51 articles should be examined. Nineteen relevant studies were identified by our search strategy [4,6-10,24-36]. Five additional studies [37-41] were found by citation searching and two studies by Google Scholar [42,43]. Ten of the 26 studies reported all required data [8-10,25,37-42]. The authors of the remaining papers were contacted for additional data. Ten authors responded [4,6,7,24,26-28,32,35,43,44] and six studies were subsequently included [4,6,7,24,26,43]. The flow diagram of our search strategy is presented in Figure 1.

### Characteristics of included studies

The sixteen studies included 3,711 patients and were carried out in a primary care setting. One study was based in the USA [39], two in Canada [4,6], one in New Zealand [38], eight in the UK [8,9,24,25,37,40,41,43] and four in other European countries [7,10,26,42]. The mean weighted prior probability is 65.1 using a reference test of ≥ 10^2 ^CFU/ml. The mean weighted prior probability using a reference test of ≥ 10^3 ^CFU/ml and ≥ 10^5 ^CFU/ml is 55.4% and 44.8% respectively. Summary characteristics of each included study are presented in Table 1.

**Table 1:** Summary of included studies

Study	Inclusion criteria	No. of patients	Mean age, y (range)	Incidence of UTI%	Reference Test	Setting and country
McIsaac 2002	Women ≥ 16 y presenting with symptoms of UTI	N =231	M = 43.9 (20-92)	53.2%	≥ 10^2 ^CFU/ml	4 urban academic family medicine clinics in Canada

McIsaac 2007	Women ≥ 16 y presenting with symptoms of UTI	N = 331	M = 45.2 (16-99)	62.8%	≥ 10^2 ^CFU/ml	225 Physicians from every province in Canada

Lawson 1973	Women presenting with symptoms of UTI	N = 343	M =? (15-55)	34.4%	≥ 10^2 ^CFU/ml	2 general practices in the UK
					≥ 10^3 ^CFU/ml	
					≥ 10^5 ^CFU/ml	

Nazareth & King 1993	Women presenting with symptoms of lower UTI	N = 54	M = 29 (16-45)	27.8%	≥ 10^2 ^CFU/ml	2 general practices in the UK
					≥ 10^3 ^CFU/ml	

Little 2006	Women with suspected UTI	N= 408	M = ? (17-70)	62.3%	≥ 10^2 ^CFU/ml	67 general practices in the UK
					≥ 10^3 ^CFU/ml	

Little 2009	Women with suspected UTI	N = 431	M = ? (17-70)	66.6%	≥ 10^2 ^CFU/ml	62 general practices in the UK
					≥ 10^3 ^CFU/ml	

Dobbs & Fleming 1987	Women presenting with symptoms of UTI	N = 238	M = ? (?)	35.7%	≥ 10^2 ^CFU/ml	3 general practices in the UK
					≥ 10^3 ^CFU/ml	

Mond 1965	Women with symptoms of UTI	N = 83	M =? (?)	45.8%	≥ 10^2 ^CFU/ml	1 general practice in the UK
					≥ 10^3 ^CFU/ml	
					≥ 10^5 ^CFU/ml	

Medina-Bombardo 2003	Women presenting with symptoms of UTI	N = 343	M = 44 (15-90)	48.4%	≥ 10^2 ^CFU/ml	18 primary health care centres in Spain
					≥ 10^3 ^CFU/ml	
					≥ 10^5 ^CFU/ml	

Dans & Klaus 1975	Women complaining of dysuria	N = 84	M = 26 (19-60)	46.4%	≥ 10^2 ^CFU/ml	Adult walk-in clinic in the US
					≥ 10^3 ^CFU/ml	

Gallagher 1965	Women with symptoms of UTI	N = 130	M=? (?)	59.2%	≥ 10^2 ^CFU/ml	Urban general practices in New Zealand
					≥ 10^3 ^CFU/ml	

Fahey 2003	Women presenting with symptoms of UTI	N = 85	M= (?)	35.7%	≥ 10^2 ^CFU/ml	8 general practices in the UK
					≥ 10^3 ^CFU/ml	
					≥ 10^5 ^CFU/ml	

Baerheim 2003	Women with acute dysuria/frequency	N = 252	M = 46 for UTI +	63.3%	≥ 10^2 ^CFU/ml	8 general practices in Norway
					≥ 10^3 ^CFU/ml	
			M = 42 for UTI-(18-87)		≥ 10^5 ^CFU/ml	

Osterberg 1996	Women with symptoms of dysuria/frequency	N= 214	M = 24 (15-35)	51.4%	≥ 10^2 ^CFU/ml	5 primary health care centres in Sweden
					≥ 10^3 ^CFU/ml	

Hummers-Pradier 2005	Women with symptoms of UTI	N = 227	M = 53 (?)	79.3%	≥ 10^2 ^CFU/ml	36 general practices in Germany
				68.7%	≥ 10^3 ^CFU/ml	
				49.3%	≥ 10^5 ^CFU/ml	

O'Brien 2007	Women with clinically suspected uncomplicated UTI	N = 111	M = 54 (?)	32.4%	≥ 10^2 ^CFU/ml	9 general practices in the UK
					≥ 10^3 ^CFU/ml	
					≥ 10^5 ^CFU/ml	

### Quality assessment

**Figure 2:**
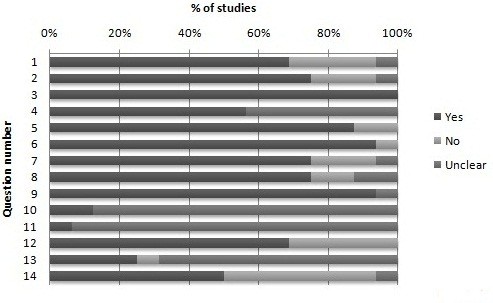
**Quality assessment**. **Included questions from the Quadas Tool**: [19] 1. Was the spectrum of patient's representative of the patients who will receive the test in practice? (Q1). 2. Were selection criteria clearly described? (Q2). 3. Is the reference standard likely to correctly classify the target condition? (Q3). 4. Is the time period between reference standard and index test short enough to be reasonably sure that the target condition did not change between the two tests? (Q4). 5. Did the whole sample or a random selection of the sample receive verification using a reference standard of diagnosis? (Q5). 6. Did patients receive the same reference standard regardless of their symptoms and signs? (Q6). 7. Were all signs and symptoms clearly defined? (Q7). 8. Was the execution of the urine culture described in sufficient detail to permit replication? (Q8). 9. Were signs and symptoms interpreted without knowledge of the results of urine culture? (Q9). 10. Were the results of the urine culture interpreted without knowledge of the symptoms and signs? (Q10). 11. Were uninterpretable/intermediate test results reported? (Q11). 12. Were withdrawals from the study explained? (Q12). **Additional question: **[20]. 13. Were the patients selected consecutively? (Q13). 14. Were statistical tests for main outcome adequate? (Q14).

The summary diagram of the quality assessment is shown in Figure 2. The overall quality of the included studies ranges from moderate to good. It is important to note that several studies were conducted before the introduction of standards for reporting diagnostic accuracy studies [37-41]. Spectrum bias is identified as a potential source of bias across certain studies, with studies including both complicated and uncomplicated patients [7,38] or failing to clearly report whether the study was focusing on complicated or uncomplicated UTI [26,40]. Partial verification bias is also noted in two studies whereby only a selected sample of patients' symptoms are verified by the reference test [24,41]. Furthermore, the presence of un-interpretable test results and blinding of symptoms and signs and reference test results are poorly reported.

### Diagnostic test accuracy of symptoms and signs

#### Summary estimates across different reference standard thresholds

Sixteen studies examined the accuracy of ten different symptoms and signs of UTI. The pooled sensitivities, specificities and the respective variance of the logit-transformed sensitivity and specificity, for individual symptoms and signs at each of the three reference standard threshold levels are presented in Tables 2, 3 and 4 respectively. Furthermore, the summary estimates of positive and negative likelihood ratio's for individual symptoms and signs at each of the three threshold levels are presented in Table 5. Six symptoms are identified as having useful diagnostic value at a reference standard threshold of ≥ 10^2 ^CFU/ml, as their 95% confidence interval values do not cross the line of no effect. Presence of dysuria, frequency, hematuria, nocturia and urgency are found to increase the probability of UTI. Presence of vaginal discharge decreases the probability of UTI. Presence of hematuria in urine has the highest diagnostic utility (LR+ 1.72), with a specificity of 0.85 and a sensitivity of 0.25, thus hematuria when present is more useful in 'ruling in' UTI. In contrast, all other significant symptoms are identified as being more useful in 'ruling out'. A similar pattern of results emerge using a higher reference standard threshold ≥ 10^3 ^CFU/ml. Consistent with lower threshold effects dysuria, frequency and urgency remain significant symptoms for ruling out a urinary tract infection at ≥ 10^5 ^CFU/ml.

**Table 2:** Summary estimates of sensitivity and specificity using a bivariate random effects model (10^2^)

SYMPTOM	No. of studies	No. of Patient	Sensitivity	(95% CI)	Variance Logit (sensitivity)	Specificity	(95% CI)	Variance Logit (specificity)
**Dysuria^a^**	14	3407	0.80	(0.74-0.86)	0.40	0.38	(0.31-0.46)	0.33
**Frequency^b^**	13	2807	0.88	(0.83-0.92)	0.41	0.20	(0.14-0.28)	0.58
**Back pain^c^**	4	635	0.38	(0.26-0.52)	0.22	0.57	(0.40-0.73)	0.43
**Fever^d^**	7	1250	0.10	(0.04-0.21)	1.19	0.92	(0.83-0.97)	1.07
**Flank pain^e^**	6	1340	0.26	(0.19-0.35)	0.21	0.69	(0.64-0.74)	0.04
**Hematuria**	7	1078	0.25	(0.21-0.29)	0.01	0.85	(0.81-0.89)	0.05
**Lower abdominal pain^f^**	7	1470	0.50	(0.34-0.66)	0.75	0.50	(0.34-0.66)	0.72
**Nocturia**	6	1720	0.59	(0.50-0.68)	0.17	0.55	(0.49-0.61)	0.06
**Urgency^g^**	9	2298	0.67	(0.52-0.80)	0.95	0.45	(0.31-0.60)	0.79
**Vaginal discharge**	6	1261	0.15	(0.08-0.26)	0.65	0.77	(0.62-0.88)	0.75

^a ^Three studies reported dysuria as painful voiding (McIsaac 2002, McIsaac 2007, Medina-Bombardo 2003)

^b ^One study reported frequency as frequency/dysuria (Dans&Klaus)

^c ^One study reported back pain as 'back or groin pain' (O'Brien)

^d ^One study reported fever as pyrexia (Lawson 1973)

^e ^One study reported flank pain as loin pain (Lawson 1973)

^f ^Different definitions were used: 'Suprapubic pain' (Hummer-Pradier 2005), Suprapubic pressure' (Baerheim), 'abdominal pain' (Fahey 2003)

^g ^One study reported urgency as urgency/frequency (Hummers-Pradier 2005)

**Table 3:** Summary estimates of sensitivity and specificity, using a bivariate random effects model (10^3^)

SYMPTOM	No. of studies	No. of Patient	Sensitivity	(95% CI)	Variance Logit (sensitivity)	Specificity	(95% CI)	Variance Logit (specificity)
**Dysuria^a^**	12	2845	0.79	(0.72-0.85)	0.39	0.39	(0.31-0.49)	0.40
**Frequency^b^**	11	2246	0.88	(0.82-0.92)	0.43	0.21	(0.14-0.31)	0.64
**Back pain^c^**	4	635	0.38	(0.26-0.52)	0.22	0.57	(0.40-0.73)	0.43
**Fever^d^**	6	926	0.12	(0.05-0.26)	1.15	0.91	(0.80-0.97)	1.16
**Flank pain^e^**	4	783	0.29	(0.18-0.43)	0.31	0.65	(0.59-0.70)	0.01
**Hematuria**	6	854	0.22	(0.18-0.27)	0.02	0.87	(0.81-0.91)	0.11
**Lower abdominal pain^f^**	5	914	0.44	(0.26-0.64)	0.78	0.58	(0.37-0.77)	0.86
**Nocturia**	5	1492	0.59	(0.48-0.70)	0.22	0.57	(0.51-0.62)	0.04
**Urgency^g^**	7	1739	0.62	(0.46-0.76)	0.72	0.51	(0.35-0.68)	0.78

^a ^One study reported dysuria as painful voiding (Medina-Bombardo 2003)

^b ^Two studies reported frequency/dysuria (Dans&Klaus, Wigton: training and validation set)

^c ^One study reported back pain as 'back or groin pain' (O'Brien)

^d ^One study reported fever as pyrexia (Lawson 1973)

^e ^One study reported flank pain as loin pain (Lawson 1973)

^f ^Different definitions were used: 'Suprapubic pain' (Hummer-Pradier 2005), Suprapubic pressure' (Baerheim), 'abdominal pain' (Fahey 2003)

^a ^One study reported urgency as urgency/frequency (Hummers-Pradier 2005)

**Table 4:** Summary estimates of sensitivity and specificity using a bivariate random effects model (10^5^)

SYMPTOM	No. of studies	No. of Patient	Sensitivity	(95% CI)	Variance Logit (sensitivity)	Specificity	(95% CI)	Variance Logit (specificity)
**Dysuria^a^**	7	1584	0.78	(0.68-0.86)	0.42	0.36	(0.26-0.48)	0.37
**Frequency**	6	1333	0.90	(0.85-0.94)	0.22	0.17	(0.11-026)	0.38
**Fever^d^**	4	742	0.10	(0.04-0.23)	0.82	0.89	(0.75-0.95)	0.79
**Lower abdominal pain^f^**	4	784	0.40	(0.21-0.62)	0.74	0.64	(0.41-0.82)	0.83
**Urgency^g^**	4	1039	0.75	(0.69-0.80)	0.05	0.36	(0.27-0.46)	0.14

^a ^One study reported dysuria as painful voiding (Medina-Bombardo 2003)

^d ^One study reported fever as pyrexica (Lawson 1973

^f ^Different definitions were used: 'Suprapubic pain' (Hummer-Pradier 2005), Suprapubic pressure' (Baerheim), 'abdominal pain' (Fahey 2003)

^g ^One study reported urgency as urgency/frequency (Hummers-Pradier 2005)

**Table 5:** Summary estimates of positive and negative likelihood ratio's, using a bivariate random effects model (10^2^, 10^3^, 10^5^)

	≥ 10^2 ^CFU/ml				≥ 10^3 ^CFU/ml				≥ 10^5 ^CFU/ml			
**SYMPTOM**	**+LR**	**(95% CI)**	**-LR**	**(95% CI)**	**+LR**	**(95% CI)**	**-LR**	**(95% CI)**	**+LR**	**(95% CI)**	**-LR**	**(95% CI)**

**Dysuria**	1.30	(1.20-1.41)	0.51	(0.43-0.61)	1.31	(1.18-1.45)	0.53	(0.43-0.64)	1.22	(1.11-1.34)	0.61	(0.50-0.74)
**Frequency**	1.10	(1.04-1.16)	0.60	(0.49-0.74)	1.12	(1.03-1.19)	0.59	(0.46-0.76)	1.09	(1.02-1.16)	0.58	(0.42-0.79)
**Back pain**	0.90	(0.71-1.14)	1.07	(0.90-1.28)	0.90	(0.71-1.14)	1.07	(0.90-1.28)				
**Fever**	1.28	(0.64-2.58)	0.98	(0.91-1.05)	1.39	(0.63-3.07)	0.96	(0.88-1.06)	0.90	(0.45-1.80)	1.01	(0.93-1.10
**Flank pain**	0.85	(0.67-1.08)	1.07	(0.98-1.17)	0.83	(0.56-1.24)	1.09	(0.92-1.29)				
**Hematuria**	1.72	(1.30-2.27)	0.88	(0.83-0.93)	1.68	(1.06-2.66)	0.89	(0.82-0.98)				
**Lower abdominal pain**	1.01	(0.89-1.15)	0.99	(0.87-1.13)	1.06	(0.88-1.29)	0.96	(0.83-1.10)	1.10	(0.87-1.38)	0.94	(0.82-1.08)
**Nocturia**	1.30	(1.08-1.56)	0.75	(0.60-0.94)	1.37	(1.13-1.65)	0.72	(0.56-0.93)				
**Urgency**	1.22	(1.11-1.34)	0.73	(0.62-0.86)	1.28	(1.11-1.47)	0.74	(0.64-0.85)	1.17	(1.04-1.31)	0.70	(0.57-0.86)
**Vaginal discharge**	0.65	(0.51-0.83)	1.10	(1.01-1.20)								

**Figure 3:**
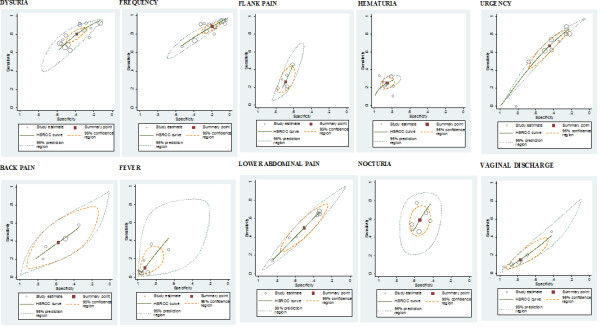
**Receiver operating characteristic graphs with 95%-confidence region and 95%- prediction region for each sign and symptom (10^2^)**.

**Figure 4:**
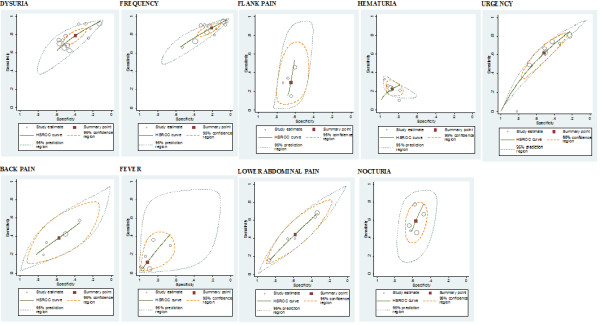
**Receiver operating characteristic graphs with 95%-confidence region and 95%- prediction region for each sign and symptom (10^3^)**.

**Figure 5:**
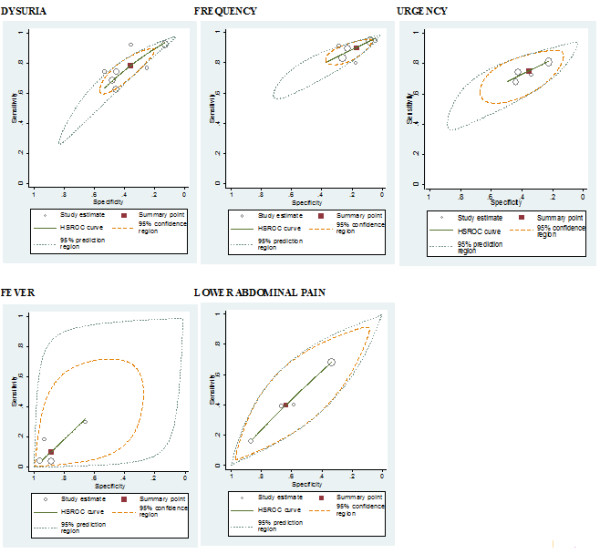
**Receiver operating characteristic graphs with 95%-confidence region and 95%-prediction region for each sign and symptom (10^5^)**.

The individual and summary estimates of sensitivity and specificity, the 95% confidence region and 95% prediction region for each symptom and sign at each of the threshold levels are presented in a receiver operating characteristic graph in figures 3, 4 and 5. The 95% confidence region remains large for several symptoms and signs across the different diagnostic thresholds, with the exception of dysuria, frequency and hematuria. This indicates greater precision of the pooled estimates for dysuria, frequency and hematuria. The 95% prediction region (amount of variation between studies) is also wide for most symptoms and signs across the different diagnostic thresholds, as reflected in the large values for the variance of logit-transformed sensitivity and specificity, with the exception of hematuria.

#### Bayesian analysis and near patient testing (dipstick)

Using Bayes theorem the post-test probability across the three threshold levels are presented in table 6. Most notable, presence of hematuria increases the pre-test probability from 65.1% to 75.8% (95% CI 70.9 - 80.1) using ≥ 10^2 ^CFU/ml and to 67.4% (95% CI 60.6 - 73.6) using ≥ 10^3 ^CFU/ml. Presence of vaginal discharge decreases the pre-test probability from 65.1% to 54.1% (95% CI 48.3 - 59.9). The probability of a UTI increases to 93.3% (≥ 10^2 ^CFU/ml) and 90.1% (≥ 10^3 ^CFU/ml) when the presence of hematuria is combined with a positive dipstick test for nitrites (table 7). Combining the presence of hematuria with a positive dipstick test for leucocyte-esterase increases the probability to 81% and 73.8% respectively (table 8). The post-test probability of UTI when the presence of dysuria, frequency, nocturia, hematuria and urgency is combined with either positive dipstick test for leucocyte-esterase or a combination of nitrites and leucocyte-esterase is also lower relative to positive symptoms combined with nitrites alone (table 8, 9). In contrast, the presence of vaginal discharge combined with a negative dipstick test result for nitrites reduces the probability of UTI to 38.4% (table 7). The presence of vaginal discharge combined with a negative result for combined nitrites and leucocyte-esterase dipstick test reduces the post-test probability further to 15% (table 9).

**Table 6:** Post-test probability of significant symptoms across three different reference standards 10^2^, 10^3 ^and 10^5 ^CFU/ml

Symptom	Reference standard*	+LR (95%CI)	Post-test probability (95%CI) (%)
**Dysuria**	10^2 ^CFU/ml	1.30 (1.20-1.41)	71.0 (70.0-71.0)
	10^3 ^CFU/ml	1.31 (1.18-1.45)	62.3 (61.0-63.6)
	10^5 ^CFU/ml	1.22 (1.11-1.34)	51.1 (49.5-52.8)
			
**Frequency**	10^2 ^CFU/ml	1.10 (1.04-1.16)	67.8 (67.0-68.5)
	10^3 ^CFU/ml	1.12 (1.03-1.19)	58.6 (57.5-59.5)
	10^5 ^CFU/ml	1.09 (1.02-1.16)	47.8 (46.7-49.0)
			
**Hematuria**	10^2 ^CFU/ml	1.72 (1.30-2.27)	75.8 (70.9-80.1)
	10^3 ^CFU/ml	1.68 (1.06-2.66)	67.4 (60.6-73.6)
			
**Nocturia**	10^2 ^CFU/ml	1.30 (1.08-1.56)	69.4 (67.3-71.3)
	10^3 ^CFU/ml	1.37 (1.13-1.65)	60.8 (58.4-63.3)
			
**Urgency**	10^2 ^CFU/ml	1.22 (1.11-1.34)	69.8 (68.5-71.1)
	10^3 ^CFU/ml	1.28 (1.11-1.47)	61.7 (59.9-63.6)
	10^5 ^CFU/ml	1.17 (1.04-1.34)	49.1 (47.1-51.1)
			
**Vaginal discharge**	10^2 ^CFU/ml	0.65(0.51-0.83)	54.1 (48.3 - 59.9)

* Pretest probability is 65.1% at ≥ 10^2 ^CFU/ml; 55.4% at ≥ 10^3 ^CFU/ml and 44.8% at ≥ 10^5 ^CFU/ml

**Table 7:** Post-test probability of significant symptoms with a positive (LR 4.42) or negative dipstick (LR 0.53) test for nitrites [23]

Symptom	Reference standard	Pre-test probability (95%CI) (%)	Post-test probability (95% CI) (LR+)	Post-test probability (95% CI) (LR-)
**Dysuria**	10^2 ^CFU/ml	71.0 (70.0-71.0)	91.5 (91.2-92.0)	56.5 (55.3-56.5)
	10^3 ^CFU/ml	62.3 (61.0-63.6)	88.0 (87.4-88.5)	46.7 (45.3-48.1)
	10^5 ^CFU/ml	51.1 (49.5-52.8)	82.2 (81.2-83.2)	35.6 (34.2-37.2)
				
**Frequency**	10^2 ^CFU/ml	67.8 (67.0-68.5)	90.3 (90.0-91.0)	52.7 (51.8-53.5)
	10^3 ^CFU/ml	58.6 (57.5-59.5)	86.2 (85.7-87.0)	42.9 (41.8-43.8)
	10^5 ^CFU/ml	47.8 (46.7-49.0)	80.2 (79.5-81.0)	32.7 (31.7-33.7)
				
**Hematuria**	10^2 ^CFU/ml	75.8 (70.9-80.1)	93.3 (91.5-95.0)	62.4 (56.4-68.1)
	10^3 ^CFU/ml	67.4 (60.6-73.6)	90.1 (87.2-92.5)	52.3 (44.9-59.6)
				
**Nocturia**	10^2 ^CFU/ml	69.4 (67.3-71.3)	91.0 (90.1-92.0)	54.6 (52.1-56.8)
	10^3 ^CFU/ml	60.8 (58.4-63.3)	87.3 (86.1-88.4)	45.1 (42.7-47.8)
				
**Urgency**	10^2 ^CFU/ml	69.8 (68.5-71.1)	91.0 (90.6-91.6)	55.1 (53.5-56.6)
	10^3 ^CFU/ml	61.7 (59.9-63.6)	87.7 (86.8-88.5)	46.1 (44.2-48.1)
	10^5 ^CFU/ml	49.1 (47.1-51.1)	81.0 (79.7-82.2)	33.8 (32.1-35.6)
				
**Vaginal discharge**	10^2 ^CFU/ml	54.1 (48.3 - 59.9)	84.0 (80.5-86.8)	38.4 (33.1-44.2)

**Table 8:** Post-test probability of significant symptoms with a positive (LR 1.36) or negative dipstick (LR 0.36) test for leucocyte-esterase [23]

Symptom	Reference standard	Pre-test probability (95%CI) (%)	Post-test probability (95%CI) (LR+)	Post-test probability (95% CI) (LR-)
**Dysuria**	10^2 ^CFU/ml	71.0 (70.0-71.0)	76.9 (76.0-76.9)	46.8 (45.7-46.8)
	10^3 ^CFU/ml	62.3 (61.0-63.6)	69.2 (68.0-70.4)	37.3 (36.0-38.6)
	10^5 ^CFU/ml	51.1 (49.5-52.8)	58.7 (57.1-60.3)	27.3 (26.1-28.7)
				
**Frequency**	10^2 ^CFU/ml	67.8 (67.0-68.5)	74.1 (73.4-74.7)	43.1 (42.2-43.9)
	10^3 ^CFU/ml	58.6 (57.5-59.5)	65.8 (64.8-66.6)	33.8 (32.8-34.6)
	10^5 ^CFU/ml	47.8 (46.7-49.0)	55.5 (54.4-56.6)	24.8 (24.0-25.7
				
**Hematuria**	10^2 ^CFU/ml	75.8 (70.9-80.1)	81.0 (76.8-84.6)	53.0 (46.7-59.2)
	10^3 ^CFU/ml	67.4 (60.6-73.6)	73.8 (67.7-79.1)	42.7 (35.6-50.1)
				
**Nocturia**	10^2 ^CFU/ml	69.4 (67.3-71.3)	75.5 (73.7-77.2)	44.9 (42.6-47.2)
	10^3 ^CFU/ml	60.8 (58.4-63.3)	67.8 (65.6-70.1)	35.8 (33.6-38.3)
				
**Urgency**	10^2 ^CFU/ml	69.8 (68.5-71.1)	75.9 (74.7-77.0)	45.4 (43.9-47.0)
	10^3 ^CFU/ml	61.7 (59.9-63.6)	68.7 (67.0-70.4)	36.7 (35.0-38.6)
	10^5 ^CFU/ml	49.1 (47.1-51.1)	56.7 (55.0-58.7)	25.8 (24.3-27.3)
				
**Vaginal discharge**	10^2 ^CFU/ml	54.1 (48.3 - 59.9)	61.6 (56.0-67.0)	29.8 (25.2-35.0)

**Table 9:** Post-test probability of significant symptoms with a positive (LR 2.57) or negative dipstick (LR 0.15) test for nitrites and leucocyte-esterase combined [23]

Symptom	Reference standard	Pre-test probability (95%CI) (%)	Post-test probability (95%CI) (LR+)	Post-test probability (95%CI) (LR-)
**Dysuria**	10^2 ^CFU/ml	71.0 (70.0-71.0)	86.3(85.7-86.3)	26.9 (25.9-26.9)
	10^3 ^CFU/ml	62.3 (61.0-63.6)	80.9(80.1-81.8)	19.9 (19.0-20.8)
	10^5 ^CFU/ml	51.1 (49.5-52.8)	72.9(71.6-74.2)	13.6 (12.8-14.3)
				
**Frequency**	10^2 ^CFU/ml	67.8 (67.0-68.5)	84.4(83.9-84.8)	24.0 (23.3-24.6)
	10^3 ^CFU/ml	58.6 (57.5-59.5)	78.4(77.7-79.1)	17.5 (16.9-18.1)
	10^5 ^CFU/ml	47.8 (46.7-49.0)	70.2(69.2-71.1)	12.1 (11.6-12.6)
				
**Hematuria**	10^2 ^CFU/ml	75.8 (70.9-80.1)	89.0(86.2-91.2)	32.0 (26.8-37.6)
	10^3 ^CFU/ml	67.4 (60.6-73.6)	84.2(79.8-87.8)	23.7 (18.7-29.5)
				
**Nocturia**	10^2 ^CFU/ml	69.4 (67.3-71.3)	85.4(84.1-86.5)	25.4 (23.6-27.1)
	10^3 ^CFU/ml	60.8 (58.4-63.3)	80.0(78.3-81.6)	18.9 (17.4-20.6)
				
**Urgency**	10^2 ^CFU/ml	69.8 (68.5-71.1)	85.6(84.8-86.3)	25.7 (24.6-27.0)
	10^3 ^CFU/ml	61.7 (59.9-63.6)	80.5(79.3-81.8)	19.5 (18.3-20.8)
	10^5 ^CFU/ml	49.1 (47.1-51.1)	71.3(69.6-72.9)	12.6 (11.8-13.6)
				
**Vaginal discharge**	10^2 ^CFU/ml	54.1 (48.3 - 59.9)	75.2 (71.0-79.3)	15.0(12.3-18.3)

## Discussion

### Principal findings

Individual symptoms and signs suggestive of a UTI have modest diagnostic discriminative value when assessed against three reference standard thresholds for UTI. Dysuria, frequency and urgency have a higher sensitivity than specificity and are more useful in ruling out a UTI diagnosis when absent across all three reference standard thresholds ≥ 10^2 ^CFU/ml, ≥ 10^3 ^CFU/ml and ≥ 10^5 ^CFU/ml. In contrast, hematuria has a higher specificity than sensitivity and is more useful in ruling in a diagnosis of UTI when present across the reference standard thresholds ≥ 10^2 ^CFU/ml and ≥ 10^3 ^CFU/ml. Combining positive dipstick test results, particularly tests for nitrites, with symptoms increases post-test probability of a UTI. In particular, presence of hematuria combined with a positive dipstick test result for nitrites increases the post-test probability from 75.8% to 93.3% at ≥ 10^2 ^CFU/ml and from 67.4% to 90.1% at ≥ 10^3 ^CFU/ml. Similarly, presence of dysuria combined with a positive dipstick test result for nitrites increases post- test probability from between 51.1% to 82.2% at ≥ 10^5 ^CFU/ml.

### Context of previous studies

The findings of this systematic review are consistent with a previous systematic review which concluded that no sign or symptom on its own is powerful enough to 'rule in' or 'rule out' the diagnosis of UTI [14]. However, the relative diagnostic importance of individual symptoms and signs varies between this review and the previous systematic review [14]. The previous systematic review found that presence of dysuria, frequency, hematuria, back pain and costovertebral angle tenderness increase the probability of UTI using a diagnostic threshold ranging from between ≥ 10^2 ^CFU/ml and ≥ 10^5 ^CFU/ml, also history of vaginal discharge, history of vaginal irritation and vaginal discharge on examination decrease the probability of a UTI. In this systematic review we found that dysuria and frequency increase the probability of UTI across different reference standard thresholds ≥ 10^2 ^CFU/ml, ≥ 10^3 ^CFU/ml and ≥ 10^5 ^CFU/ml. Hematuria is also significant in the present study using a diagnostic threshold of ≥ 10^2 ^CFU/ml and ≥ 10^3 ^CFU/ml. However, in contrast to the previous systematic review back pain is not significantly associated with UTI across the different reference standard thresholds. Vaginal discharge is identified as an important symptom for decreasing the probability of UTI in the present study.

Such differences may be an artefact of different methodological approaches taken. Firstly, the previous systematic review pooled all studies irrespective of the reference standard threshold used, whereas the present study sought to determine the importance of individual symptoms and signs at different reference standard thresholds. In addition, our inclusion criteria was more conservative, excluding studies which involved self-diagnosis, case-control study designs and different healthcare settings (i.e. not primary care settings) where the prevalence of symptoms may differ and increase the chance of spectrum bias.

### Strengths and limitations of this study

The systematic search, the conservative inclusion criteria, the inclusion of additional data from authors, and the quality assessment of the included studies can be seen as strengths of this study. In addition, given the lack of consensus regarding reference standard thresholds for UTI, the current study is the first study to determine the diagnostic accuracy of symptoms and signs across the three thresholds ≥ 10^2 ^CFU/ml, ≥ 10^3 ^CFU/ml and ≥ 10^5 ^CFU/ml. Lastly, this study highlights the additional importance of using dipstick test, particularly tests for nitrites, as an additional diagnostic tool when ruling in a UTI diagnosis based on particular symptomatology.

We acknowledge that this review has limitations. Variability of diagnostic accuracy estimates across studies is high. This may be due to the fact that we did not restrict the age of women included in the meta-analysis. It is known that the prevalence of UTI differs across age groups, peaking at 15-24 years and greater than 65 years [45]. In addition definitions used to describe individual symptoms and signs vary across studies. For example, 'lower abdominal pain' has been defined as 'suprapubic pain'[4,6,7], 'suprapubic pressure' [42] or 'abdominal pain' [24]. Furthermore, as the bivariate random effects model is used symptoms and signs are analysed, when at least 4 studies are included. Therefore few symptoms and signs are excluded from our meta-analysis particularly at the higher reference standard threshold of ≥ 10^5 ^CFU/ml. Finally, while the probability of UTI increases when the presence of certain symptoms are combined with positive dipstick test results, it is important to acknowledge that the predictive value of the dipstick test result, was taken from a meta-analysis which included men and pregnant women [23].

### Implications for practice

Individual symptoms and signs will modestly increase the post-test probability and cannot accurately 'rule in' or 'rule out' the diagnosis of a UTI. Subgroup analysis shows improved diagnostic accuracy using lower reference standards of ≥ 10^2 ^CFU/ml and of ≥ 10^3 ^CFU/ml. In addition, combining nitrite dipstick test results with clinical symptoms and signs is useful for ruling in a UTI diagnosis and deciding on the optimal patient management strategy. More recently, formal clinical prediction rules for UTI that incorporate the independent effects of symptoms and signs into a "risk score" have been proposed as an alternative management strategy when considering antibiotic treatment [8]. This approach appears to be equivalent to alternative management strategies for UTI in women including empirical immediate antibiotics, empirical delayed antibiotics, or use of near-patient testing with a dipstick in terms of duration or severity of symptoms. However, in terms of antibiotic usage, use of a dipstick results in fewer antibiotics being prescribed when compared to immediate empirical antibiotics or use of a UTI "risk score" [46].

### Future studies

The current approach of evaluating symptoms and signs as a diagnostic test is in general two-dimensional, and ignores symptom severity [8,9,28] In the future, focusing on severity of symptoms may provide more valuable diagnostic information.

## Conclusions

Individual symptoms and signs, independent of reference standard threshold have a modest ability to 'rule in' or 'rule out' the diagnosis of UTI. Use of a dipstick test enhances diagnostic utility and reduces the chance of prescribing unnecessary antibiotics. Future studies should focus on the refinement of diagnosis utilising information on severity and duration of symptoms, alone, in combination and alongside dipstick testing.

## Competing interests

The authors declare that they have no competing interests.

## Authors' contributions

All authors were involved in the study conception and design. LG performed a systematic search of the literature. Both LG and GC screened potential articles, evaluated the methodological quality of studies, acquired data for analysis, performed statistical analysis and interpretation of data and drafted the paper. TF, FVL and BDD critically revised the draft manuscript. All authors read and approved the final manuscript.

## Pre-publication history

The pre-publication history for this paper can be accessed here:

http://www.biomedcentral.com/1471-2296/11/78/prepub

## Supplementary Material

Additional file 1 **PRISMA checklist**. Guidelines for the reporting on systematic reviews and meta-analyses Click here for file
